# The McMaster Health Information Research Unit: Over a Quarter-Century of Health Informatics Supporting Evidence-Based Medicine

**DOI:** 10.2196/58764

**Published:** 2024-07-31

**Authors:** Cynthia Lokker, K Ann McKibbon, Muhammad Afzal, Tamara Navarro, Lori-Ann Linkins, R Brian Haynes, Alfonso Iorio

**Affiliations:** 1 Health Information Research Unit Department of Health Research Methods, Evidence, and Impact McMaster University Hamilton, ON Canada; 2 Department of Computing and Data Science Birmingham City University Birmingham United Kingdom; 3 Department of Medicine McMaster University Hamilton, ON Canada

**Keywords:** health informatics, evidence-based medicine, information retrieval, evidence-based, health information, Boolean, natural language processing, NLP, journal, article, Health Information Research Unit, HiRU

## Abstract

Evidence-based medicine (EBM) emerged from McMaster University in the 1980-1990s, which emphasizes the integration of the best research evidence with clinical expertise and patient values. The Health Information Research Unit (HiRU) was created at McMaster University in 1985 to support EBM. Early on, digital health informatics took the form of teaching clinicians how to search MEDLINE with modems and phone lines. Searching and retrieval of published articles were transformed as electronic platforms provided greater access to clinically relevant studies, systematic reviews, and clinical practice guidelines, with PubMed playing a pivotal role. In the early 2000s, the HiRU introduced Clinical Queries—validated search filters derived from the curated, gold-standard, human-appraised Hedges dataset—to enhance the precision of searches, allowing clinicians to hone their queries based on study design, population, and outcomes. Currently, almost 1 million articles are added to PubMed annually. To filter through this volume of heterogenous publications for clinically important articles, the HiRU team and other researchers have been applying classical machine learning, deep learning, and, increasingly, large language models (LLMs). These approaches are built upon the foundation of gold-standard annotated datasets and humans in the loop for active machine learning. In this viewpoint, we explore the evolution of health informatics in supporting evidence search and retrieval processes over the past 25+ years within the HiRU, including the evolving roles of LLMs and responsible artificial intelligence, as we continue to facilitate the dissemination of knowledge, enabling clinicians to integrate the best available evidence into their clinical practice.

## History of the Heath Information Research Unit

The McMaster University School of Medicine was founded in 1967. One of its basic principles, and arguably one of its most important, was using problems and experience in clinical settings (ie, problem-based learning [PBL]) for health sciences education rather than reliance on lectures and expert opinions. Probably the biggest challenge of PBL was how to identify and apply the best current evidence-based knowledge from the medical literature to address clinical problems.

**Figure 1:**
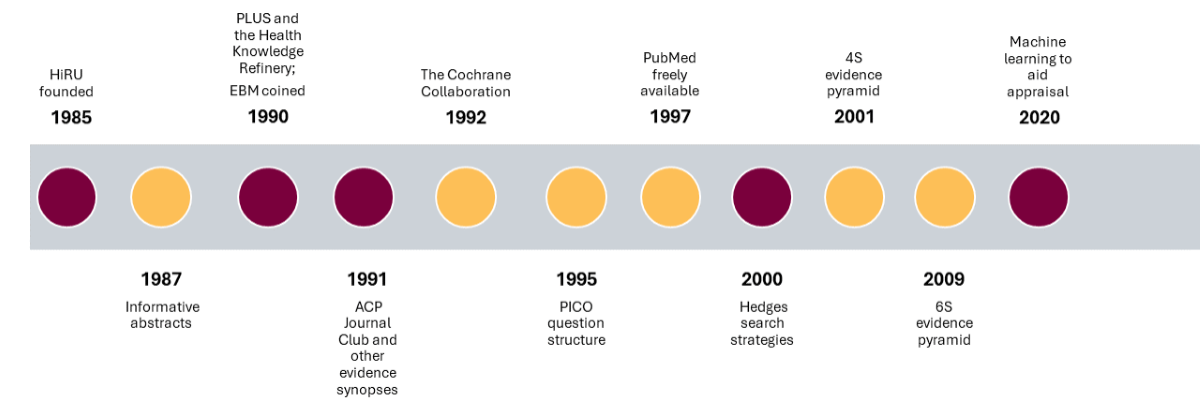
Key milestones for the Health Information Research Unit (HiRU; maroon) and evidence-based medicine (EBM; yellow). ACP: American College of Physicians; PICO: patient/population, intervention, comparator, outcome; PLUS:Premium LiteratUre Service.

The Health Information Research Unit (HiRU) was formed in 1985 (Figure 1) with funding from the Rockefeller Foundation. Since the HiRU started, we have been working on the problem of providing the best evidence from studies to the clinicians who need it quickly, efficiently, and in easy-to-use formats. Over the years, we have researched and developed tools to achieve this goal, building in a stepwise fashion. Our first set of studies centered on evaluating dissemination and utilization methods [1], now often referred to as knowledge translation. As we worked on the task of integrating research findings into practice, we realized that finding and evaluating studies that were ready for clinical practice were greater challenges than we first thought.

In the early 1980s, David Sackett and his colleagues at McMaster University published a series of articles in the *Canadian Medical Association Journal* on how to read a clinical journal article [2]. In 1991, Gordon Guyatt at McMaster University coined the phrase “evidence-based medicine” (EBM) [3]. This was followed by publication of the “Users’ guides to the medical literature” in the *Journal of the American Medical Association* [4,5]. These guides offered an influential series of articles that were well received and changed approaches to medical decision-making in times of uncertainty while keeping current with changing practice. The EBM movement grew; however, the problem of having fast and efficient access to the best literature remained.

A parallel step in efficient access and understanding came when Brian Haynes, founder of the HiRU, and colleagues from around the world petitioned medical journal editors to require more informative abstracts for clinically important articles. They stated that clinical studies could be more readily appraised (by aiding rapid comprehension) if abstracts, which were freely and widely available via MEDLINE, included the information that was needed for both critical appraisal of scientific merit and appropriate clinical use. They proposed that the 200-300–word abstracts include the exact question addressed, study design, findings directly pertinent to the study question, and key conclusions for clinical application [6,7]. This structure has been adopted by most clinical journals. Notably, the resulting structured abstract has been an aid for researchers in the fields of natural language and artificial intelligence (AI) interested in retrieving and summarizing studies. Along the same line, the HiRU designed and delivered *ACP Journal Club* in collaboration with the American College of Physicians (ACP) [8]. Each month, highly structured summaries of high-quality, high-impact articles across a list of core clinical journals are published in *Annals of Internal Medicine* with an accompanying commentary by a practicing clinician. Worth noting, other universal paradigms of EBM stemmed from editorials published in *ACP Journal Club* and *Evidence-Based Medicine*. In 1995, an editorial discussed the value of structuring clinical questions using the main components (ie, population/patient, intervention, comparator, and outcome [PICO] terms) [9], and a series of editorials published between 2001 and 2016 described the evolving 4S, 5S, and 6S hierarchies or pyramids as models for organizing and selecting the best available evidence [10].

Despite these advances, finding studies with the best evidence for real-time clinical care remained a challenge. The HiRU broached training clinicians to search MEDLINE [11,12]. If a clinician could easily search the literature, they might be more likely to practice EBM. This and similar studies found that while clinicians could learn to search MEDLINE, the search strategy development process was cumbersome and time-consuming. The HiRU helped the US National Library of Medicine (NLM) by developing and testing early versions of Grateful Med [13]. This software program made searching MEDLINE easier, especially for clinicians. In 1997, PubMed became a free, searchable database that includes the abstracts and records in MEDLINE, which was visited by an average 3.4 million users each day in 2021 [14].

## Innovations in Search Retrieval and Preappraised Evidence Resources for Clinicians

### Hedges

With funding from the Canadian Institutes of Health Research and the US National Institutes of Health/NLM, we next started analyzing index and abstract terms in MEDLINE to see if we could identify (and then build) “canned” searches that would retrieve only the studies with the strongest methods (eg, randomized controlled trials for studies of treatment). This would allow for content searches to be limited to articles with a higher likelihood of being rigorously performed. We were successful with this endeavor and have continuously conducted research to improve these “clinical queries” using advanced information analysis and retrieval methods, including new work with AI.

To accomplish this, we developed a method to evaluate the performance of Boolean search terms and combinations, termed “hedges,” to retrieve target articles, which represents an early natural language processing application. The method is based on a manual search of 160 clinical journals for the year 2000, with 49,028 articles classified by article format, interest to human health care, and purpose category, which were critically appraised by highly trained staff with expertise in health research methods. The resulting Hedges dataset was used to test thousands of combinations of search terms using a diagnostic accuracy test approach [15]. The Hedges strategies retrieve original and review articles [16] for a range of purposes such as treatment [17], prediction guides [18], etiology [19], prognosis [20], and diagnosis [21]. Database-specific strategies are available on our website [22], with several available through PubMed, MEDLINE, and EMBASE as Clinical Queries. In 2013, we reported on the robustness, assessed over 10 years, of the strategies for retrieving relevant articles in PubMed [23].

### McMaster PLUS

**Figure 2:**
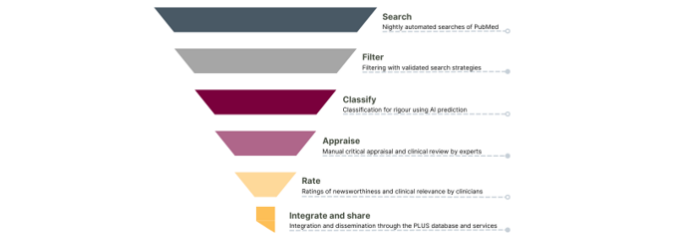
Health Information Research Unit Health Knowledge Refinery process. AI: artificial intelligence; PLUS: Premium LiteratUre Service.

McMaster PLUS (Premium LiteratUre Service) curates high-quality evidence and is comprised of a series of steps in a process referred to as the Health Knowledge Refinery (HKR) (Figure 2). The HKR was designed to distill the flow of articles from a broad selection of clinical journals into a refined product of preappraised literature to support clinicians through several evidence services and products.

The current PLUS process integrates several HiRU innovations. First, nightly automated searches of ~125 journals (selected from a critical appraisal of >800 clinical journals) are filtered using highly sensitive Hedges search strategies developed by the unit in the early 2000s to identify systematic reviews, evidence-based guidelines, and original studies addressing questions of treatment, prevention, quality improvement, economics, diagnosis, etiology, prediction guides, and prognosis.

Second, the filtrate is further refined using a recently developed BioBERT (Bidirectional Encoder Representations from Transformers for Biomedical Text Mining)–based machine learning model that classifies articles for meeting explicit criteria for research rigor and clinical relevance. The model maintains 99% sensitivity (recall) and high precision, reducing the work required to manually appraise articles by 60% [24].

Third, research associates manually critically appraise the filtered articles using established standards for scientific rigor. From a list of 63 clinical disciplines, they select the relevant medical disciplines and indicate if the article is also relevant to rehabilitation professions or nursing. Article assessments are then reviewed by a clinician with expertise in research methods.

Fourth, the McMaster Online Rating of Evidence (MORE) system provides postpublication clinical peer review to further refine the HKR output to the articles that are important for consideration in clinical practice [25]. Qualifying articles are automatically sent to registered clinicians for each pertinent discipline identified during the critical appraisal step. MORE raters are a crowd of ~6000 health care providers practicing worldwide, who rate the articles on 7-point scales for relevance to their practice and newsworthiness (defined as useful new information for physicians). Physicians [26], nurses [27], and rehabilitation practitioners [28] are invited to join MORE via specific links.

Fifth, the final refined HKR filtrate of articles that have relevance and newsworthiness scores ≥4 is sent out via email alerts; added to the PLUS database; and made available through searchable interfaces such as ACP JournalWise, Evidence Alerts, ACCESSSS, and other products and services to support a range of clinical knowledge users (Table 1). We provide content to publishers for updating evidence-based textbooks, which we customize under the auspices of McMaster University, a not-for-profit, publicly funded university. Usage of HiRU products remains strong. Across several services, we have >250,000 registered users with >25,000 new registrations in 2023.

Finally, through PLUS, selected articles that are highly rated for clinical relevance and newsworthiness to practicing clinicians and that are of interest to the broad readership of *Annals of Internal Medicine* are summarized and included in *ACP Journal Club*. This EBM-focused enterprise has been active since 1990 and has evolved over time. Originally, article selection was done through a manual search of the contents of printed journals by trained research associates with articles that met the methods criteria shared through fax machines. This process evolved to reviewing the online table of contents and sharing through email. In 2014, we developed an in-house web-based infrastructure that allowed for automated retrieval of article titles and abstracts from PubMed, a collection of a range of data elements entered by research associates during critical appraisal, automated email-based alerting for clinical editors and MORE raters, and a collection of ratings. This also allowed us to track articles that were not applicable to the HKR (eg, not related to human health care, basic science, and methodology studies), those that were relevant but did not meet methodologic criteria, and those below clinical relevance and newsworthiness thresholds for alerting. Through this process, we have curated a dataset of almost 200,000 articles that have been reviewed by human experts, along with various associated data elements gathered at the time of publication.

**Table 1:** Current McMaster PLUS (Premium LiteratUre Service) projects and alerting services [29].

Project or service	Description
EvidenceAlerts	Alerting service for physicians, nurses, and rehabilitation professionals
ACCESSSS	A smart search engine that retrieves content from multiple sources and orders it according to the pyramid of evidence
ACP^a^ Journal Club	Synopses with accompanying clinical commentaries of high-quality, clinically relevant studies, published monthly in *Annals of Internal Medicine*
ACP JournalWise	Alerting service and platform for searching and filtering from the top 120 clinical journals with options to personalize content
DynaMed	Alerting service customized for DynaMed editors and authors
EBM Guidelines	Alerting service for Duodecim editors and authors
Pain+	Alerting service for pain specialists
Rehab+	Alerting service for rehabilitation professionals
Public Health+	Alerting service for public health professionals
CLOT+	Alerting service for hematology/thrombosis
KT+	Alerting service for knowledge translation researchers and workers
McMaster Optimal Aging Portal	Alerting service for practitioners, patients, and the public interested in evidence relevant to aging
STAT!Ref Evidence Alerts	Alerting service customized for Teton Data Systems
Helsebiblioteket.no	ACCESSSS customized for the Norwegian National Health library

^a^ACP: American College of Physicians.

## The Evolving Role of AI and Machine Learning

### The Era of Machine Learning

The Hedges dataset provided the HiRU with opportunities to collaborate with external research partners. The dataset has served as a validated reference standard of high-quality studies and has been used to build and test more advanced literature retrieval models; the search strategies have also been used to identify articles to build comparison datasets. Historically, the retrieval models included conventional methods such as Boolean search filters and citation-based algorithms. Over time, machine learning approaches, particularly supervised models trained using these data, have been effective in retrieving high-quality clinical studies from the biomedical literature [30]. Some examples include the study by Aphinyanaphongs et al [31] in 2005, which used articles abstracted in *ACP Journal Club* (as a PLUS derivative) and machine learning to automatically construct filters identifying high-quality, content-specific articles in internal medicine. Building on this work, using the Hedges dataset, Kilicoglu et al [32] experimented with 3 supervised machine learning methods (naïve Bayes, support vector machine, and boosting), and obtained comparatively better results.

Advancements in machine learning have dramatically improved computer capabilities through deep neural networks. In 2018, Del Fiol et al [33] used the Hedges dataset to develop a model that was perhaps the first to investigate the use of deep learning techniques to identify reports of scientifically sound studies in the biomedical literature. In 2020, Afzal et al [34] used an optimized multilayer feed-forward neural network model, the multilayer perceptron, for identifying scientifically sound studies and filtering out others. Both studies used the PubMed Clinical Queries filter with a “narrow” scope, favoring high precision over high recall [33,34].

Through the HKR, our dataset of classified and appraised articles has grown, and we have expanded our machine learning capabilities. We recently used the data to train machine learning models in-house, achieving both high recall (sensitivity) and precision. We assessed the efficacy of advanced deep learning models that include BERT (Bidirectional Encoder Representations from Transformers) and its variations such as BioBERT, BlueBERT, and PubMedBERT [35]. In 2023, a state-of-the-art model named DL-PLUS, trained using the dataset, excelled in classifying articles for meeting rigor and clinical relevance compared with competitors [24]. This represents our initial phase of machine learning work, with plans to improve the classification of articles by purpose category (eg, treatment, diagnosis) and rigor to provide more targeted results for searchers.

### Large Language Models

The incorporation of LLMs into health care is rapidly growing, and EBM is no exception. LLMs have demonstrated remarkable success in various downstream tasks, including information extraction and evidence summarization of clinical studies and bodies of literature [36,37]. To enable the uptake of evidence into practice, LLMs have potential to summarize individual studies and bodies of evidence. At the HiRU, we have conducted pilot testing of AI-generated summaries to gauge how well they include EBM-pertinent details such as a clear research objective, details on the methodology, effect sizes, and conclusions for clinical application. Analogous to our early focus on ensuring ready access to interpretable findings and identifying the best-quality research, of key interest is developing and testing prompts (much like search queries) that task the AI tools with returning the necessary information and assessing the factuality of generated summaries. Our near-future plans include contributing to the methods of ensuring the trustworthiness of evidence summaries generated by LLMs [38], with a focus on a clinical audience. Our extended plan includes developing custom LLMs at the HiRU, contributing to other tasks of the evidence ecosystem such as evidence appraisals.

### Responsible AI

The majority of machine learning models, particularly deep learning models, are inherently “black boxes,” concealing internal details about the decision-making processes [39]. As a result, determining the true effectiveness of an AI model becomes challenging. In some cases, bias may inherently exist in the dataset (eg, it does not accurately represent the overall population), and machine learning engineers may unintentionally introduce bias (eg, during class balancing through sampling). In a recent model training experiment, we observed that when we attempted to balance classes with an oversampling method, the result was a highly accurate yet poorly calibrated model. Subsequent evaluations using calibration methods revealed that the model with the initial unbalanced data was, in fact, well-calibrated. Therefore, responsible AI practice is crucial, particularly in digital health applications, due to the potential ethical issues associated with AI technologies. The increasing reliance on AI in health care has highlighted the importance of addressing concerns such as biases, discrimination, errors, and lack of transparency in outcomes. Implementing responsible AI practices in this context becomes paramount, emphasizing ethical principles and human values to minimize biases, enhance fairness, ensure interpretability, and ultimately prevent adverse consequences on human and societal well-being [40]. Our focus on responsible AI for futuristic AI models will target 2 key areas: prioritizing the examination of datasets to identify biases related to coverage, correctness, and fairness, while concurrently enhancing the interpretability and explainability of AI model prediction workflows, thereby clarifying the decision-making process.

## Conclusion

Over the last quarter-century, the HiRU has been at the leading edge of developing novel processes and tools to support clinicians in the practice of EBM, which is work that continues to support a wide range of users and clients. The mission of the HiRU is to harness information science and technology to build customized, high-efficiency, continuously updated evidence services. The fast pace of change in machine learning and AI in recent years is providing a new landscape for innovation. With collaborators across disciplines, we plan to leverage new and emerging tools to continue to facilitate health care evidence retrieval, appraisal, and dissemination while building on our foundation of ensuring the quality and trustworthiness of the work we develop.

## Abbreviations

ACP: American College of Physicians
AI: artificial intelligence
BERT: Bidirectional Encoder Representations from Transformers
BioBERT: Bidirectional Encoder Representations from Transformers for Biomedical Text Mining
EBM: evidence-based medicine
HiRU: Health Information Research Unit
HKR: Health Knowledge Refinery
LLM: large language model
MORE: McMaster Online Rating of Evidence
NLM: Natural Library of Medicine
PBL: problem-based learning
PICO: population/patient, intervention, comparator, and outcome
PLUS: Premium LiteratUre Service
